# Early detection and management of mesenteric traction syndrome using the hypotension prediction index: a case report

**DOI:** 10.1186/s40981-025-00838-0

**Published:** 2025-12-02

**Authors:** Rui Yajima, Takayuki Sugai, Kei Inoue, Takashi Ouchi, Toshiya Koitabashi

**Affiliations:** Department of Anesthesiology, Tokyo Dental College Ichikawa General Hospital, 5-11-13, Sugano, Ichikawa City, Chiba, 272-8513 Japan

**Keywords:** Hypotension prediction index, Mesenteric traction syndrome, Perioperative monitoring, Artificial intelligence, Intraoperative hypotension

## Abstract

Mesenteric traction syndrome (MTS) is a transient circulatory disturbance characterized by sudden hypotension, tachycardia, and facial flushing following mesenteric manipulation during abdominal surgery. Early recognition is essential; however, conventional monitoring often detects these events only after hemodynamic deterioration. We report a 68-year-old man who underwent distal pancreatectomy and splenectomy under combined general and thoracic epidural anesthesia. Sixteen minutes after the start of surgery, the hypotension prediction index (HPI)—an artificial intelligence–based monitoring system—abruptly increased to > 85 despite stable vital signs. The systemic vascular resistance index decreased, suggesting early vasodilatory change. Prompt intravenous phenylephrine boluses were administered, and rapid colloid infusions were initiated to minimize hypotension. Recognition of facial flushing subsequently confirmed the diagnosis of MTS, and intravenous flurbiprofen stabilized the circulation. This case suggests that HPI monitoring may assist in the early identification of vasodilatory changes associated with MTS and support timely intervention before severe hypotension develops.

## Background

Mesenteric traction syndrome (MTS) is a transient circulatory disturbance that typically presents with hypotension, tachycardia, and facial flushing shortly after mesenteric manipulation during abdominal surgery [1]. It is believed to result from the release of vasodilatory mediators, such as prostacyclin, from the mesentery, leading to a rapid decrease in systemic vascular resistance [2, 3]. In some cases, transient histamine release has also been reported as a contributing factor [4]. Although MTS usually resolves spontaneously, it can occasionally cause profound hypotension that requires prompt treatment.

Conventional monitoring often detects MTS only after hypotension has already developed, limiting opportunities for preventive intervention. The Hypotension Prediction Index™ (HPI) is a machine learning–based algorithm that analyzes arterial pressure waveforms to predict hypotension several minutes before its clinical onset [5, 6]. The HPI ranges from 0 to 100, with values greater than 85 indicating a high likelihood of hypotension developing within the next few minutes. According to the manufacturer’s recommendation, an HPI value of 85 is defined as the predictive cutoff for impending hypotension. Randomized trials have demonstrated that HPI-guided management can reduce both the severity and duration of intraoperative hypotension [5]. However, its usefulness in detecting MTS has rarely been reported. We present a case in which HPI monitoring enabled early recognition of MTS and timely intervention, thereby minimizing the severity of hypotension.

## Case presentation

A 68-year-old man (height, 168 cm; weight, 66 kg) was scheduled to undergo distal pancreatectomy and splenectomy. His medical history included hypertension and glaucoma. General anesthesia was induced with propofol 70 mg and remifentanil 0.4 µg/kg/min, followed by rocuronium 60 mg to facilitate tracheal intubation. Anesthesia was maintained with sevoflurane (end-tidal concentration, 1.0%) in air–oxygen and a continuous remifentanil infusion of 0.15 µg/kg/min. Depth of anesthesia was monitored using the bispectral index, and neuromuscular function was continuously assessed with quantitative train-of-four stimulation.

In addition to standard monitoring, a radial arterial catheter connected to an Acumen IQ™ sensor was used for HPI monitoring. Hemodynamic data were recorded and displayed using the HemoSphere™ Advanced Monitoring Platform (all devices from Becton, Dickinson and Company, Irvine, CA, USA). All parameters were updated every 20 s and displayed as time-series trends, with each value representing the mean of the preceding 20 s.

A thoracic epidural catheter was placed at the T8–9 interspace, and 5 mL of 0.25% levobupivacaine was administered 15 min before skin incision. At the time of incision, mean arterial pressure (MAP) was 93 mmHg, pulse rate (PR) 45 beats/min, systemic vascular resistance index (SVRI) 2739 dyn·s/cm5·m2, and HPI 15.

**Fig. 1:**
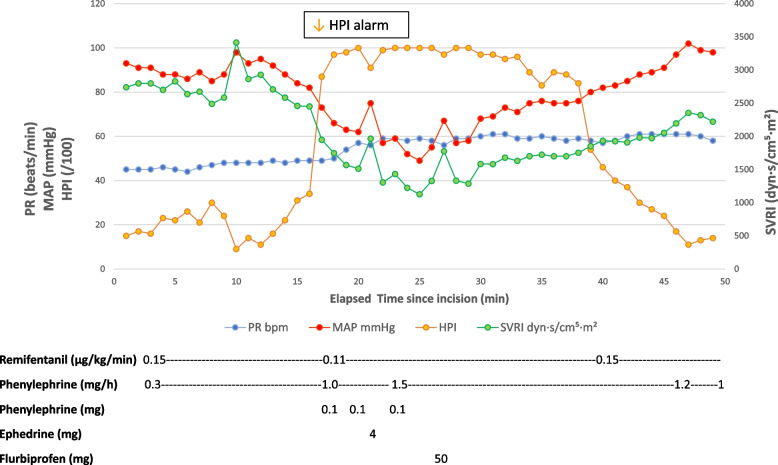
Hemodynamic changes and therapeutic interventions during the development of mesenteric traction syndrome. Time-course changes in the hypotension prediction index (HPI), mean arterial pressure (MAP), systemic vascular resistance index (SVRI), and pulse rate (PR) are shown. Sixteen minutes after skin incision, the HPI increased concurrently with a decrease in SVRI, prompting therapeutic intervention before critical hypotension occurred. Despite rapid infusion of colloid solution and administration of phenylephrine, hypotension persisted, requiring additional boluses of phenylephrine and ephedrine. Intravenous flurbiprofen 50 mg was administered 11 min after the initial HPI alarm (HPI > 85), after which hemodynamic stability was promptly restored. The timing and dosage of each therapeutic intervention are also indicated in the figure. A downward arrow indicates the timing of the first HPI alarm (HPI > 85)

Sixteen minutes later, although MAP (73 mmHg) and PR remained stable, the HPI rose abruptly from 44 to 87 over a 20-s update interval, triggering an audible alarm. The time course of hemodynamic parameters and the timing of therapeutic interventions are illustrated in Fig. 1. At this point, SVRI had already decreased from 2255 to 1948 dyn·s/cm5·m2. Intravenous phenylephrine 0.1 mg was administered immediately, the continuous phenylephrine infusion was increased from 0.3 to 1.0 mg/h, and rapid colloid infusion was initiated. The remifentanil infusion rate was reduced from 0.15 to 0.11 µg/kg/min.

Despite these interventions, after 3 min, SVRI declined further to 1512 dyn·s/cm^5^·m^2^, MAP decreased to 62 mmHg, PR rose slightly to 57 beats/min, and the HPI reached 100. Two additional 0.1 mg boluses of phenylephrine were administered, the infusion rate was increased to 1.5 mg/h, and a 4 mg dose of ephedrine was given. The lowest arterial pressure was recorded 6 min after the initial HPI alarm, when the MAP was 51 mmHg, PR 59 beats/min, SVRI 1156 dyn·s/cm^5^·m^2^, and HPI 100.

The cumulative duration of MAP < 55 mmHg was approximately 100 s. Facial flushing was observed approximately 10 min after the initial HPI alarm when the drape was lifted, confirming the diagnosis of MTS. Intravenous flurbiprofen 50 mg was then administered, after which blood pressure and SVRI stabilized promptly, and no further hypotensive episodes attributable to MTS occurred.

## Discussion

In this case, a transient episode of MTS occurred during distal pancreatectomy and splenectomy. The HPI increased markedly before hypotension became evident, prompting early treatment with vasopressors and colloid infusion. This case illustrates how HPI monitoring can facilitate the early recognition and management of vasodilatory hypotension associated with MTS. Early identification and timely intervention are crucial because the vasodilation accompanying MTS can rapidly lead to hypotension. In this case, the HPI provided an early warning before overt hemodynamic deterioration, enabling preventive intervention.

The HPI is a machine learning–based algorithm developed from over 130 million arterial waveforms, using 23 hemodynamic features and logistic regression to detect early instability and estimate the probability of hypotension before its clinical onset [6, 7]. Although there is no universally accepted definition of intraoperative hypotension, the HPI algorithm defines a hypotensive event as a MAP < 65 mmHg sustained for at least one minute. In the present case, the HPI exceeded 85 before any clinically evident change in arterial pressure or increase in heart rate, coinciding with an early reduction in systemic vascular resistance. This allowed earlier administration of vasopressors and fluids, thereby reducing both the severity and duration of hypotension.

Although the HPI exceeded the alert threshold, the SVRI at that moment remained within the normal range (1,948 dyn·s/cm^5^·m^2^). A slight downward trend was present, but the change was subtle and may not have been readily recognized in routine clinical practice. Because SVRI typically decreases only after vasodilation has progressed, identifying early instability based solely on SVRI can be challenging. In contrast, the HPI incorporates 23 subtle waveform-derived features and updates every 20 s, enabling more sensitive detection of early hemodynamic instability before measurable hypotension develops.

While MTS was ultimately confirmed in this case, other causes of intraoperative hypotension—particularly vasodilatory etiologies such as hypovolemia, bleeding, anesthetic overdose, or anaphylaxis, as well as cardiogenic causes such as acute heart failure or myocardial depression—should also be considered when the HPI rises. Therefore, HPI should be regarded as a predictive aid rather than a diagnostic tool and should be interpreted in conjunction with clinical findings and the surgical context.

The Perioperative Quality Initiative recommends maintaining MAP ≥ 65 mmHg to reduce the risk of myocardial and renal injury [8]. Large observational studies have shown that even brief exposure to MAP < 55 mmHg is independently associated with postoperative cardiac and renal complications [9–13]. In our patient, the cumulative duration of MAP < 55 mmHg was limited to approximately 100 s, suggesting that exposure to potentially harmful hypotension remained minimal.

This experience also highlights the importance of familiarity with predictive monitoring systems. Because this was an early clinical use of HPI in our institution, initial hesitation in responding to the alarm delayed the definitive diagnosis of MTS by approximately 10 min. Appropriate clinical use of HPI requires not only understanding its numerical thresholds but also recognizing its limitations, potential differential diagnoses, and the need for integration with other hemodynamic parameters.

Although an elevated HPI indicates an increased probability of impending hypotension, it does not distinguish whether the underlying mechanism is hypovolemia, vasodilation, or a cardiogenic cause. Therefore, HPI should be interpreted together with other hemodynamic variables, such as SVRI, stroke volume, stroke volume variation, and contractility. Importantly, the HPI platform also provides additional indices that allow clinicians to consider potential mechanisms of circulatory instability—including hypovolemia as a form of preload reduction, decreased afterload, or impaired contractility. These data can assist in guiding therapeutic decisions, although final interpretation always requires clinical judgment.

In our view, HPI monitoring is particularly valuable in patients undergoing major surgery who are at increased risk of hemodynamic instability, such as those with cardiovascular or renal comorbidities in whom intraoperative hypotension may lead to serious perioperative complications. Because the device is reimbursable in Japan only for patients who meet the criteria for continuous noninvasive cardiac output monitoring, its use should be guided by both surgical invasiveness and individual patient risk rather than by surgical category alone.

This case suggests that HPI monitoring may assist in the early recognition of vasodilatory changes associated with MTS and facilitate timely hemodynamic intervention before severe hypotension develops. Further studies and cumulative clinical experience are needed to clarify its role in different perioperative settings and vasodilatory pathologies.

## Data Availability

Not applicable.

## Abbreviations

HPI: Hypotension prediction index
MAP: Mean arterial pressure
MTS: Mesenteric traction syndrome
PR: Pulse rate
SVRI: Systemic vascular resistance index
